# Rapid Detection of *Escherichia coli* O157 and Shiga Toxins by Lateral Flow Immunoassays

**DOI:** 10.3390/toxins8040092

**Published:** 2016-03-25

**Authors:** Jinliang Wang, Robab Katani, Lingling Li, Narasimha Hegde, Elisabeth L. Roberts, Vivek Kapur, Chitrita DebRoy

**Affiliations:** Department of Veterinary and Biomedical Sciences, The Pennsylvania State University, 115 Henning Building, University Park, PA 16802, USA; wjl478@163.com (J.W.); rxk104@psu.edu (R.K.); lul17@psu.edu (L.L.); hegdenv@yahoo.com (N.H.); elr2@psu.edu (E.L.R.); vkapur@psu.edu (V.K.)

**Keywords:** lateral flow immunoassay (LFIA), colloidal gold, *E. coli* O157, Shiga toxin, Stx, STEC, food safety

## Abstract

Shiga toxin-producing *Escherichia coli* O157:H7 (STEC) cause food-borne illness that may be fatal. STEC strains enumerate two types of potent Shiga toxins (Stx1 and Stx2) that are responsible for causing diseases. It is important to detect the *E. coli* O157 and Shiga toxins in food to prevent outbreak of diseases. We describe the development of two multi-analyte antibody-based lateral flow immunoassays (LFIA); one for the detection of Stx1 and Stx2 and one for the detection of *E. coli* O157 that may be used simultaneously to detect pathogenic *E. coli* O157:H7. The LFIA strips were developed by conjugating nano colloidal gold particles with monoclonal antibodies against Stx1 and Stx2 and anti-lipid A antibodies to capture Shiga toxins and O157 antigen, respectively. Our results indicate that the LFIA for Stx is highly specific and detected Stx1 and Stx2 within three hours of induction of STEC with ciprofloxacin at 37 °C. The limit of detection for *E. coli* O157 LFIA was found to be 10^5^ CFU/mL in ground beef spiked with the pathogen. The LFIAs are rapid, accurate and easy to use and do not require sophisticated equipment or trained personnel. Following the assay, colored bands on the membrane develop for end-point detection. The LFIAs may be used for screening STEC in food and the environment.

## 1. Introduction

Worldwide, a large number of foodborne outbreaks are attributable to the consumption of contaminated food due to Shiga toxin-producing *Escherichia coli* (STEC) [1,2,3]. The clinical spectrum of STEC-associated human disease varies considerably, from diarrhea to hemorrhagic colitis (HC), to life threatening hemolytic uremic syndrome (HUS), particularly in children and elderly. While a large fraction of reported STEC infections is attributable to *E. coli* O157:H7, six serogroups (O26, O45, O103, O111, O121, and O145) account for approximately 70% of non-O157 STEC infections in the United States [3]. Recently, Food Safety and Inspection Services of the U.S. Department of Agriculture have declared these STEC strains to be adulterants in meat [4]. Since cattle are the primary reservoirs of STEC, it is now required that all ground meat samples be tested for STEC O-groups and Shiga toxins (Stx) as these organisms are shed in feces and contaminate the environment for prolonged period of time [5]. Because of regulations imposed, STEC can cause significant economic loss, especially to the beef industry, due to product embargoes, voluntary destruction of product, and nationwide product recalls. STEC strains produce potent Shiga toxins (Stx), a family of related toxins, which is composed of two major toxin types, Stx1 and Stx2, that have 56% homologous nucleotide sequence and are grouped into several allelic types [6]. Stx2 is more potent and have been found to be associated with strains that have caused outbreak of diseases [7]. The *stx* genes are encoded by prophages, and are induced by antibiotics such as ciprofloxacin [8]. Stx may also play a role by inducing mucosal inflammation due to stimulation of proinflammatory cytokines by epithelial cells [9]. The toxins have an active *N*-4 glycosidase A-subunit associated with five identical B-subunits. Because Stx is highly potent, the presence of *stx* genes is tested routinely for distinguishing the virulent strains from commensal strains for epidemiological studies, outbreak investigations and food monitoring. *E. coli* O157 and other STEC strains are detected by microbiological procedures and multiplex PCR [10,11,12,13]. The process takes a long time, as the cells have to be grown for several hours before testing can be conducted. While many of the strains may carry the *stx* gene, the expression of Shiga toxins varies considerably. The immunological techniques currently available [14,15,16,17,18] for the detection of O-groups and Stx1 and 2 require knowledgeable scientists to carry out the experiments and cannot be applied for rapid on-site detection. In the present study, we report the development of two simple and sensitive lateral flow devices that can detect *E. coli* O157 and Stx1 and 2 rapidly and accurately without using sophisticated instrumentation. The lateral flow immunoassay (LFIA), is a solid-phase immunoassay, combining the principles of thin layer chromatography and immune recognition reaction that has been effectively used in many fields [19]. They are low cost, user-friendly and stable in a wide range of applications, for the detection of pathogens and diseases [20,21,22,23,24,25,26,27], and various environmental and agricultural contaminations [28,29,30,31]. In this paper, we describe the development of two easy to use multi-analyte antibody-based LFIAs, one for the detection of Stx1 and 2 and another for *E. coli* O157 that may be used simultaneously for detecting pathogenic *E. coli* O157. The LFIA devices can be used with little training and require only an assessment of colored lines on the membrane for end-point detection.

## 2. Results and Discussion

**Figure 1:**
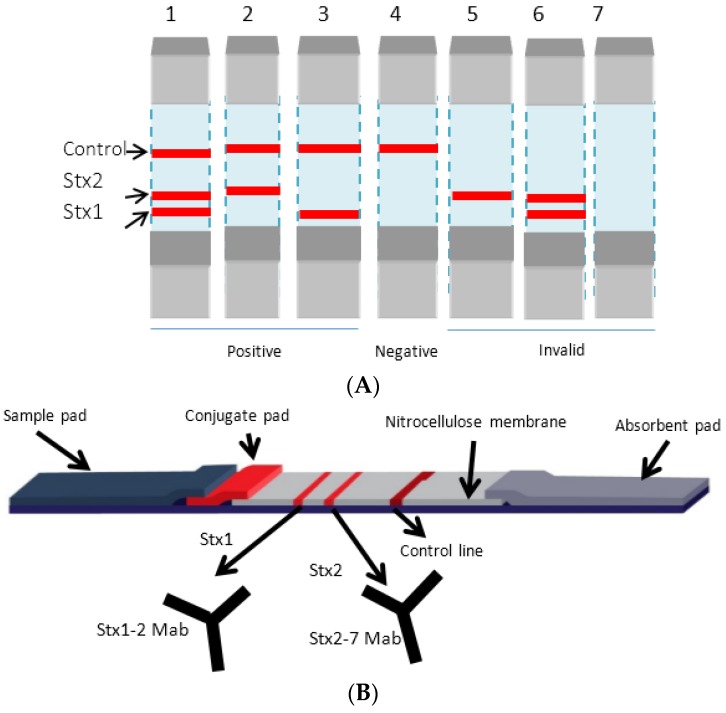
Diagrammatic representation of Lateral Flow Immunoassay Device. (**A**) The results expected to be generated following assay (**B**) The LFIA device.

The LFIAs described were developed such that the target antigen (Stx or *E. coli* O157), when present in the sample, will be captured by colloidal gold-labeled antibody in the conjugation pad, would migrate to the test line and generate a red signal following the formation of antigen-antibody complex in the nitrocellulose membrane as diagrammatically represented in Figure 1. Test lines would exhibit red color indicating the presence of Stx1 and/or Stx2 (Figure 1(A1–A3)). The intensity of the color depicted in test lines would be proportional to the amount of Shiga toxin in the sample. When the target molecule in the sample is below the detection limit, the test signal will not appear showing the result to be negative (Figure 1(A4)). If the control line does not form, the test is invalid (Figure 1(A5–A7)).

**Figure 2:**
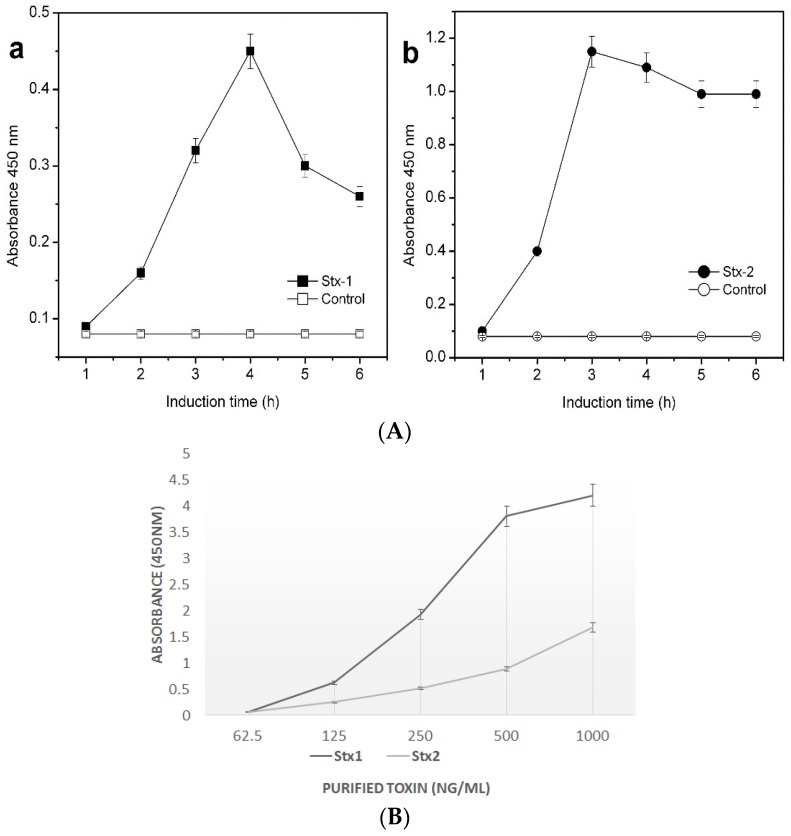

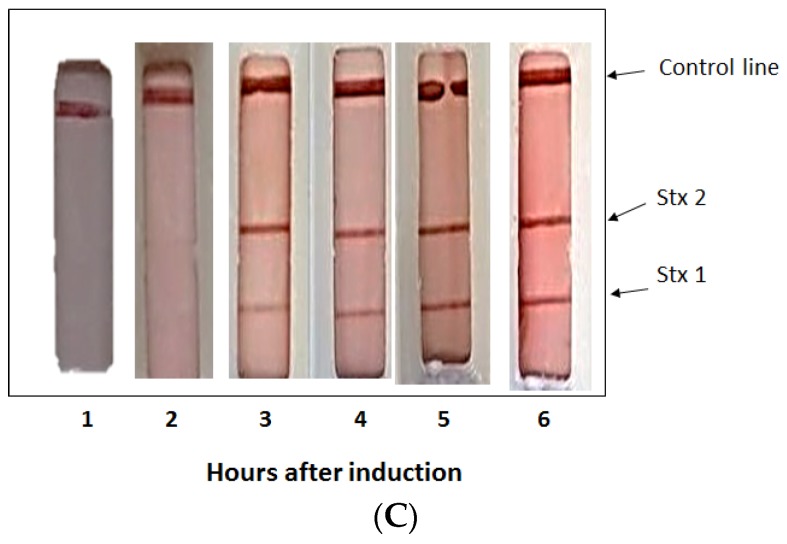
Comparison of Stx ELISA and LFIA. (**A**) ELISA for the detection of Stx1 (**a**) and Stx2 (**b**) following 1 to 6 h of induction of *E. coli* O157:H7; (**B**) Standard Curve to quantify Stx1 and Stx2; (**C**) Detection of Stx1 and 2 by LFIA of *E. coli* O157 following 1 to 6 h of induction.

Detection of Stx1 and 2 by ELISA and LFIA: Following induction of *E. coli* O157:H7 with ciprofloxacin, production of Stx1 and Stx2 were assessed after different time periods using ELISA (Figure 2A). Based on the standard curve generated for quantifying of Stx1 and Stx2 expressed, using pure toxins (Figure 2B), Stx1 production was found to be maximum (about ~90 ng) after 4 h of induction by ELISA (Figure 2(Aa)). Stx2, on the other hand, was found to be expressed more (~700 ng) after 3 h of induction in ELISA (Figure 2(Ab)). Using LFIA and ELISA, Shiga toxin (Stx1 and 2) production could be detected after 3 h of induction. The LFIA test strips could detect both Stx1 and Stx2 simultaneously as colored bands as shown in Figure 2C. The specificity of LFIA was evaluated by using strains that produced Stx, such as STEC belonging to *E. coli* O26, O45, O103, O111, O121, O145 and O157 *versus* those that did not produce any Stx as determined by the absence of *stx* genes by PCR (Table 1). The results indicated that the strains that produced Stx1 and Stx2 exhibited colored lines at the expected positions in LFIA, whereas there were no lines observed for the *stx* negative strains, while both STEC and non-STEC strains exhibited the control lines reflecting the tests were valid. Some of the Stx2 producing strains were found to carry *stx*2 variant genes *stx*2a and *stx*2c as shown in Table 1. The Shiga toxins produced by these strains could be detected by both ELISA and LFIA reflecting the antibodies were effective against some of the alleles of Stx2 reflecting high sensitivity and specificity (Table 2).

**Figure 3:**
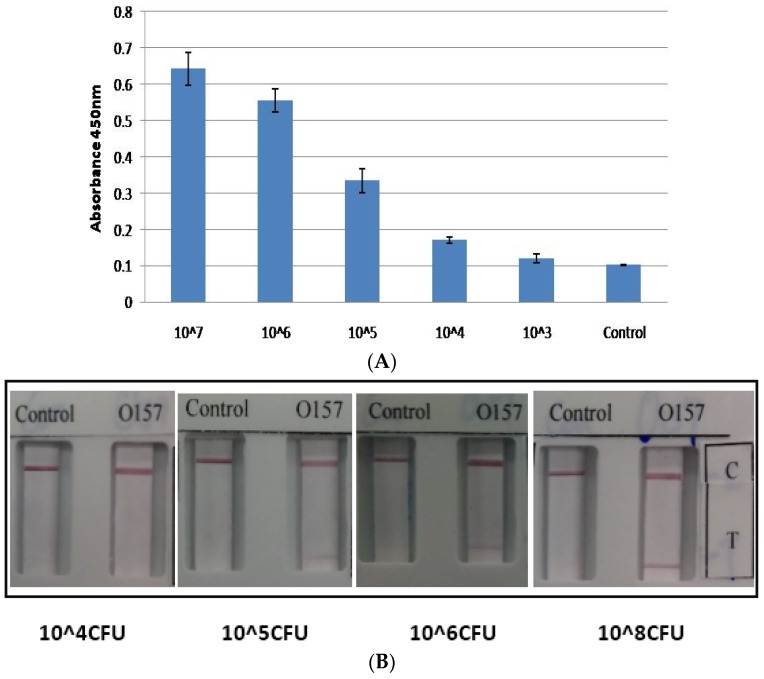
Comparison of *E. coli* O157 ELISA and LFIA. Bacteria were grown in LB media and detected at different concentrations. (**A**) ELISA for the detection of *E. coli* O157 at different concentrations; (**B**) LFIA at different concentrations of *E. coli* O157 (10^4^ CFU/mL, 10^5^ CFU/mL, 10^6^ CFU/mL and 10^8^ CFU/mL). Control “C”: Negative-non-*E. coli* O157 at 10^8^ CFU/mL; “T”: *E. coli* O157.

ELISA and LFIA for *E. coli* O157 was also found to be consistent and the limit of detection (LOD) was found to be 10^5^ CFU/mL for both ELISA and LFIA (Figure 3A,B). The ELISA and LFIA were found to be highly sensitive and specific when tested against *E. coli* O157 positive and negative strains (Table 2). The specificity against all non-O157 O groups, as well as non-*E. coli* strains listed in Material and Methods were also tested.

The LFIA of Stx and *E. coli* O157 in beef spiked with 10 CFU/mL of *E. coli* O157 and grown overnight as described in methods, exhibited positive results 3 h after induction at a bacterial concentration of 10^5^ CFU/mL.

Currently, there are some commercial products available for *E. coli* O157 and Stx that are similar to the ones described in this paper, but the cost of the products is expensive. Recently, lateral flow assays for Shiga toxins [21] and *E. coli* O157 [20] were reported. LFIA for Stx described [21], used several monoclonal antibodies for Stx1 and Stx2 that captured both Stx1 and Stx2 epitopes, and was not designed to distinguish the two types, Stx1 and Stx2. The LFIA described for *E. coli* O157 [20] which is signal-amplified is more sensitive (1.14 × 10^3^ CFU/mL). The LFIA for O157, described in this report, has utilized antibodies to lipid A as the capture antibody for *E. coli*, making it novel that has not been used by other investigators. Since lipid A is highly conserved, this method can be used to develop LFIA for other STEC O groups. The two LFIAs for the detection of both *E. coli* O157 and Stx may be used simultaneously to detect pathogenic *E. coli* O157. With the development of highly specific antibodies against Shiga toxins and *E. coli* O157, more sensitive LFIA could be developed in the future. The LFIAs developed in this report, were found to be very accurate with no false negative or false positive results making this method very useful for screening Stx and *E. coli* O157 in food and the environment. The LFIAs were rapid tests that took only a few minutes to exhibit colored reactions, are simple to use and did not require expensive equipment or trained personnel to interpret the results. These LFIA devices therefore, may be used to detect Stx and *E. coli* O157 in samples from various sources like water, meat or any other food that require screening for STEC. Rapid detection technology is an important tool in the fight against food-borne disease. Unlike ELISA or PCR methods, the colloidal gold technology can be used for point-of-care applications and screening as they require only assessment of colored red lines for end-point detection.

## 3. Materials and Methods

Bacteria: Bacterial strains used for the study were from the culture collection of the *E. coli* Reference Center at the Pennsylvania State University. To validate the specificity of the Stx LFIA, clinical isolates of STEC belonging to serogroups O26 (*n* = 5), O103 (*n* = 5), O111 (*n* = 3), O121 (*n* = 2), O145 (*n* = 2) and O157 (*n* = 6) and control strain *E. coli* K12 (*n* = 1) (Table 1) that were characterized for the presence of *stx*1 and 2 genes by PCR were included. For assessing the specificity of *E. coli* O157 LFIA, *E. coli* O157 (*n* = 10) and 174 *E. coli* standard reference strains were utilized that belonged to serogroups O1-O181, excluding O31, O47, O67, O72, O94, and O122, since these serogroups have not been designated [32]. Ten strains representative of other bacterial genera, *Citrobacter freundii*, *Enterobacter cloacae*, *Hafnia alvei*, *Klebsiella pneumoniae*, *Proteus vulgaris*, *Salmonella enterica sv Enteritidis and Salmonella enterica sv Typhi*, *Serratia marcescens*, *Shigella boydii*, and *Shigella flexneri* were also used to test the specificities of both Stx and O157 LFIAs. All of the bacteria were grown in Luria Bertani (LB) broth or on LB agar plates at 37 °C.

Preparation of Shiga toxin: Single colony of *E. coli* O157:H7 strain was inoculated in LB medium and incubated at 37 °C overnight on a rotating shaker set at 150 rpm. Overnight-grown culture was inoculated into fresh LB medium (5 mL) containing ciprofloxacin (100 ng/mL) (Fluka, Ronkonkoma, NY, USA) to a final concentration of OD_600_ ~0.2. The culture was grown at 37 °C for 1–6 h with continuous shaking. The cell culture was harvested by centrifugation at 6000× *g* for 10 min and the cell supernatant (100 μL) was passed through 0.2 μm hydrophobic polytetrafluoroethylene syringe filter (Millipore Corporation, Billerica, MA, USA) and used for the detection of Shiga toxin. The supernatant was stored at −20 °C until further used. For detecting *E. coli* O157:H7, the cultures were serially diluted in phosphate buffered saline (PBS) for enumeration. Antigens were derived from bacteria (at concentrations 10^8^, 10^6^, 10^5^ and 10^4^ CFU/mL) by boiling for 30 min at 100 °C and the heat-inactivated antigen (100 μL) was utilized for the detection. The same procedure was employed for generating antigens from all bacteria.

Antibodies: Four purified monoclonal antibodies (MAbs), designated as Stx1-1, Stx1-2, Stx2-5 and Stx2-7, kindly donated by Dr. Xiaohua He, Western Regional Research Center, USDA, Albany, CA, USA [17] were used for the development LFIA devices. Rabbit anti-mouse IgG and bovine serum albumin fraction V were purchased from Roche (Roche Diagnostics, Indianapolis, IN, USA), mouse monoclonal antibody to lipid A was purchased from Abcam (Cambridge, MA, USA) and rabbit anti-mouse IgG peroxidase conjugate was purchased from Sigma (Sigma-Aldrich, St. Louis, MO, USA).

Purification of the rabbit anti-O157 IgG: *E. coli* O157 antigen was prepared as described earlier [14] and injected in New Zealand rabbits by Cocalico Biologicals Inc. (Reamstown, PA, USA). The IgG was purified from antisera using NAb™ Spin Kits (Pierce Biotechnology, Rockford, IL, USA) according to manufacturer’s protocol. Concentration of purified IgG was determined in a spectrophotometer (Nanodrop, Wilmington, DE, USA) at 280 nm and by SDS-polyacrylamide gel electrophoresis.

Detection of Stx and *E. coli* O157 by ELISA: The sandwich ELISA for Stx was performed as described previously with modifications [15]. Polystyrene microtiter plates were coated with 100 µL of ceramide trihexoside (2.5 µg/mL) (Matreya, Bellefonte, PA, USA) in methanol that was allowed to evaporate overnight at room temperature. Wells were filled with 200 μL of blocking solution (4% BSA in 0.01 M phosphate buffered saline (PBS) containing 0.05% Tween-20) and incubated at 4 °C overnight, followed by two washes with distilled water. The plates were stored at −20 °C until use. After induction with ciprofloxacin, bacterial cell supernatants containing Shiga toxin (100 μL) were dispensed in triplicate wells and incubated with shaking at 37 °C for 1 h, followed by washing three times with 200 µL distilled water. Primary antibodies generated against for Stx1 or Stx2 (Santa Cruz Biotechnology, Dallas, TX, USA) were diluted (1 µg/mL) in 0.01 M PBS containing 4% BSA, 0.05% Tween-20. Diluted antibodies (100 μL) were dispensed in each well and incubated for 1 h at 37 °C. The wells were washed three times with 200 μL of distilled water after removing the contents of the wells. Secondary antibody-peroxidase conjugate (1 µg/mL) (100 μL), prepared in 0.01 M PBS containing 4% BSA and 0.05% Tween-20, was added to each well and the plates were incubated for 1 h at 37 °C. The washing step was repeated and finally, one-step ultra TMB (100 µL) was added to each well and the plates were incubated for 15 min at room temperature. An equal volume of stopping solution (1 M HCl) was added and the optical density was read at 450 nm using a plate reader. Wells containing all components of the assay but without antigen, served as negative controls for determining the background noise. The background OD_450_ ranged from 0.071 to 0.103. All background readings were subtracted, and the averages of triplicate readings were resolved. An OD_450_ of 0.29–0.3 above the background was considered positive for Shiga toxins. The assays were repeated three times and the average is presented. Detection of *E. coli* O157 by ELISA was carried out following our laboratory protocol described earlier [14].

Preparation of colloidal gold: Colloidal gold was prepared according to the method described [31] with some modifications. Gold suspension HAuCl_4_·3H_2_O (2 mL of 1% solution) (Sigma Chemical Co., St. Louis, MO, USA) was added to boiling deionized water (200 mL) and heated to 100 °C for 4 min, 1% sodium citrate (2 mL) solution was then added rapidly with constant stirring. The solution changed its color from pale yellow to brilliant red, which indicated complete reduction of the gold chloride. The solution was kept at 100 °C for another 10 min, the heating mantle was then removed, and stirring was continued for 15 min until the solution reached room temperature. Distilled water was then added to make the total volume of 200 mL. The average particle diameter was ~40 nm as checked in spectrophotometer.

Conjugation of antibodies and colloidal gold: The colloidal gold nanoparticles were conjugated to antibodies, Stx1-1 and Stx2-5 by optimizing the pH and determining the concentrations of antibodies for optimal binding. Different volumes of 0.1 M K_2_CO_3_ were added to 1 mL colloidal gold suspension and 12 µL volume was judged to be optimum for conjugation. Different concentrations of antibodies were added and 6 µg of MAb was found to be optimum as determined by spectrophotometric analysis of the suspension in the absorbance range 450 nm to 600 nm. The conjugates were eventually blocked by 1% bovine serum albumin (BSA) for 30 min at room temperature. The labeled conjugate was centrifuged at 1000× *g* for 5 min and the supernatant further centrifuged at 4 °C for 15 min at 12000× *g*. The pellet containing the colloidal gold particle conjugated with antibodies, was resuspended in one tenth of initial volume of a solution Tris-HCl (0.05 M, pH 8.0) containing BSA (2%) and PEG 20000 (0.5%) and stored at 4 °C.

Immobilization of reagents: Stx1-2, Stx2-7 MAbs and rabbit anti-O157 IgG (detection antibodies) were diluted to 1 mg/mL concentrations with PBS (pH 7.4) and used for printing the test line to detect antigen. The polyclonal rabbit anti-mouse IgG antibody (Sigma Chemical Co.) diluted to 0.5 mg/mL with PBS (pH 7.4) was used as the control line. The optimal conditions for printing the antibodies were found to be optimum at 6 volts at a speed of 0.35 mL/minute. The gap between the two test lines for Stx1 and Stx2 was designed to be 0.4 cm and the gap between the test line and the control line was designed to be 0.6 cm. These antibodies were printed by lateral flow reagent dispenser on a nitrocellulose membrane (NC) (Claremont Biosolutions, Upland, CA, USA) following the manufacturer’s protocol using different voltage and different rates of flow. Following printing the NC membranes were dried at room temperature.

Fabrication of LFIA: The strip was composed of three pads, sample, conjugate and absorbent and NC membrane. They were pasted to an adhesive plastic backing plate 4 mm wide. The NC membrane containing immobilized antibodies was pasted in the center of the backing plate. Long strips (10 cm × 0.5 cm) of glass fiber conjugate pads were dipped in 250 μL of gold-labeled capture antibody solutions and dried for 1 h at 37 °C. The conjugate pad containing gold-labeled antibodies were pasted by overcrossing 2 mm with the NC membrane. The sample pad was also pasted by over-crossing 4 mm with conjugate pad. The absorbent pad was pasted on the other side of the plate. The whole assembled unit was 5 × 70 mm in dimension.

Sensitivity and specificity of the assays for Shiga toxins and *E. coli* O157: The sensitivity of the assay was determined by detecting Stx generated from *E. coli* O157:H7 and other STEC strains (Table 1) that were induced for different time periods by ELISA and compared with LFIA. The quantity of the Stx produced by ELISA was assessed in standard curves generated by absorbance at 460 nm with pure Stx1 and Stx2 toxins at different concentrations. The assays were repeated three times. The specificity of the assays was examined by detecting the Stx produced by STEC and non-STEC strains (Table 1) by ELISA and compared with LFIA. The limit of detection, defined as the minimum CFU required to produce a signal in ELISA and LFIA for *E. coli* O157 was determined at different concentrations of the bacteria (10^8^, 10^6^, 10^5^, and 10^4^ CFU/mL). Antigens were prepared from cultures at each dilution, and assays were performed as described [14]. The experiment was repeated three times.

Detection of *E. coli* O157 and Shiga toxin in artificially inoculated ground beef samples: Ground beef (25 g) samples purchased from a local store were individually spiked with 1–10 CFU of *E. coli* O157:H7 and propagated in 250 mL of Tryptic Soy Broth medium. All samples were pre-enriched by incubating static for 6 h at 37 °C in the presence of vancomycin (16 mg/L). Following pre-enrichment, the cultures were supplemented with rifampicin (2 mg/L), and potassium tellurite (1 mg/L), and incubation continued for 18 h at 42 °C on a shaker. An un-inoculated ground beef sample was enriched similarly and used as control. Subsequently, the samples were passed through qualitative filter paper, the bacteria (1 mL) were harvested by centrifugation at 6000× *g* for 10 min, re-suspended in PBS (1 mL) and boiled for 30 min at 100 °C to generate antigens, and 100 μL of supernatant was used for detecting Shiga toxin.

## Figures and Tables

**Table 1:** Bacterial strains used in this study.

Serial #	*Bacteria*	ID#/ECRC #	Serotype	*stx*1	*stx*2 (Type)	Expression of Stx
1	*E. coli*	0.1304	O157:H7	+	−	+
2	*E. coli*	90.2281	O157:H7	+	−	+
3	*E. coli*	96.0428	O157:H7	+	−	+
4	*E. coli*	0.1288	O157:H7	+	+ (a,c)	+
5	*E. coli*	0.1292	O157:H7	+	+ (a)	+
6	*E. coli*	3.0190	O157:H7	+	−	+
7	*E. coli*	0.1302	O26:NM	+	−	+
8	*E. coli*	5.2217	O26:H11	+	−	+
9	*E. coli*	7.3964	O26:H11	+	−	+
10	*E. coli*	8.0176	O26:H30	+	−	+
11	*E. coli*	77.0044	O26:HNM	+	−	+
12	*E. coli*	10.2529	O103:H2	+	−	+
13	*E. coli*	6.1623	O103:H36	+	+ (a,c)	+
14	*E. coli*	3.2605	O103:H2	+	−	+
15	*E. coli*	9.0108	O103:H2	+	−	+
16	*E. coli*	85.1983	O103:H21	+	−	+
17	*E. coli*	0.0320	O111:H?	+	−	+
18	*E. coli*	0.1079	O111:NM	+	−	+
19	*E. coli*	0.1481	O111:H?	+	−	+
20	*E. coli*	5.0959	O121:H19	−	+ (a)	+
21	*E. coli*	7.1636	O121:H19	−	+ (a)	+
22	*E. coli*	6.1598	O145:H+	−	+ (a)	+
23	*E. coli*	95.1167	O145:HNM	−	+ (c)	+
24	*E. coli*	K12		−	−	−
25	*Citrobacter freundii*	ATCC 8090		−	−	−
26	*Enterobacter cloacae*	15.0057		−	−	−
27	*Hafnia alvei*	ATCC 29926		−	−	−
28	*Klebsiella pneumoniae*	ATCC 27736		−	−	−
29	*Proteus vulgaris*	15.0061		−	−	−
30	*Salmonella enterica sv Enteritidis*	15.0060		−	−	−
31	*Salmonella enterica sv Typhi*	15.0056		−	−	−
32	*Serratia marcescens*	ATCC 13880		−	−	−
33	*Shigella boydii*	15.0055		−	−	−
34	*Shigella flexneri*	15.0059		−	−	−

#: Number; +: Positive; −: Negative.

**Table 2:** Diagnostic sensitivity and specificity of the assays.

Assay	Status	Reference Specimen of Known Status		Sensitivity	Specificity
		Positive (*n*)	Negative (*n*)		
Stx1 and Stx2	Positive	23	0	23/23 = 100%	11/11 = 100%
	Negative	0	11		
O157	Positive	10	0	10/10 = 100%	184/184 = 100%
	Negative	0	184		
